# In vitro and in vivo antitumor effects of the Egyptian scorpion *Androctonus amoreuxi* venom in an Ehrlich ascites tumor model

**DOI:** 10.1186/s40064-016-2269-3

**Published:** 2016-05-10

**Authors:** Mohamed L. Salem, Nahla M. Shoukry, Wafaa K. Teleb, Mohamed M. Abdel-Daim, Mohamed A. Abdel-Rahman

**Affiliations:** Zoology Department, Faculty of Science, Tanta University, Tanta, Egypt; Zoology Department, Faculty of Science, Suez University, Suez, Egypt; Pharmacology Department, Faculty of Veterinary Medicine, Suez Canal University, Ismailia, 41522 Egypt; Zoology Department, Faculty of Science, Suez Canal University, Ismailia, 41522 Egypt

**Keywords:** *Androctonus amoreuxi*, Apoptosis, Cancer, Egypt, Scorpion, Venom, Ehrlich ascites carcinoma

## Abstract

Scorpion venom is a highly complex mixture of about 100–700 different components, where peptides are the major constituents with various biological and pharmacological properties including anticancer activities. In this study, anticancer efficacy of the venom of the Egyptian scorpion *Androctonus amoreuxi* has been evaluated. In vitro, the human breast cancer MCF-7 cell line was treated with the venom and the IC_50_ was estimated. In vivo studies, Ehrlich ascites carcinoma (EAC) cells were inoculated into CD-1 mice intraperitoneally to form liquid tumor or subcutaneously to form solid tumor and then treated with intraperitoneal injection with venom (0.22 mg/kg) every other day. The total tumor cells in the ascitic fluid and the size of the solid tumor were assessed after 14 and 30 days, respectively. In addition, the mean survival time (MST), body weight, tumor volume, PCV, viability of tumor cells, CBC, AST, ALP, creatinine, oxidative stress biomarkers (GSH, MDA, PCC), tumor marker Ki67, growth factor VEGF and caspase-3 were measured in normal control, EAC control and venom-treated groups (n = 6). Treatment with venom induced anti-tumor effects against liquid and in solid tumors as indicated by a significant (*P* < 0.05) reduction in tumor volume/size, count of viable EAC cells, expression of Ki67 and VEGF as well as by remarkable increases in MST and caspase-3 expression as compared to non-treated group. Interestingly, the venom restored the altered hematological and biochemical parameters of tumor-bearing animals and significantly increased their life span. These data indicate to (1) the cytotoxic potential effects of *A. amoreuxi* on tumor cells via anti-proliferative, apoptotic and anti-angiogenic activities; (2) opening a new avenue for further studies on the anti-cancer effects of this agent.

## Background

According to the reports and statistics from the International Agency for Research on Cancer (IARC) and the World Health Organization (WHO), cancer has become the leading cause of deaths worldwide (7.6, 8.2 million deaths in 2008 and 2012, respectively) (Jemal et al. 2011; Ferlay et al. 2013). However chemotherapy and radiotherapy are extensively being used to treat several types of tumors, they have many disadvantages such as (1) deleterious side effects on normal cells (lack of specificity); (2) low success rate and (3) high risk of recurrence. Consequently, development of new safe and effective strategies for cancer diagnosis and treatment is extremely needed. One of these alternative approaches is using anticancer peptides derived from animal venoms including scorpions. Various previous studies revealed effectiveness of the crude scorpion venoms and certain purified peptides (such as iberiotoxin margatoxin, charybdotoxin, AGAP-SYPU2 and chlorotoxin) against cancer cells through different mechanism of actions (Deshane et al. 2003; Bloch et al. 2007; Liu et al. 2009; Jang et al. 2011; Zhao et al. 2011; Shao et al. 2014; Ding et al. 2014; Ortiz et al. 2015).

Scorpion venom is a cocktail of protein (enzymes and peptides) and non-protein (inorganic salts, lipids, nucleotides, free amino acids and water) substances produced by the venom gland for defense and prey capture (Rodriguez de la Vega and Possani 2005; Abdel-Rahman et al. 2015). Disulfide-bridged peptides and non-disulfide-bridged peptides constitute the major groups of scorpion venom and many of them have been characterized with analgesic, anti-epileptic, hemolytic, anti-thrombotic, anti-inflammatory, antimicrobial and anticancer activities (Yu et al. 1992; Wang et al. 2001; Zeng et al. 2005; Song et al. 2005; Shao et al. 2007; Mamelak 2011; Harrison et al. 2014, 2016; Almaaytah and Albalas 2014; Abdel-Rahman et al. 2016).

The anticancer efficacy of scorpion venoms has been examined in various types of cancers as glioma, neuroblastoma, leukemia, lymphoma, breast, lung, prostate and pancreatic cancer (Ding et al. 2014). Only a few toxins have been found to be the responsible for these anticancer effects and exert their action by three different mechanisms: (1) blocking specific ion channels (Jager et al. 2004); (2) inhibiting invasion and metastasis of cancer cells (Deshane et al. 2003); (3) activating intracellular pathways leading to cell cycle arrest and apoptosis (Gupta et al. 2010). Recently, venom from the Buthidae scorpions *Androctonus bicolor*, *Androctonus crassicauda*, and *Leiurus quinquestriatus* (collected from Saudi Arabia) revealed strong anticancer activity on colorectal and breast cancer cell lines (HCT-116 and MDA-MB-231, respectively) through decreasing cell motility and colony formation of cancer cells (Al-Asmari et al. 2016).

However several scorpion species (about 24 species) are widespread in Egypt, only few studies highlighted anticancer activity of scorpion venoms such as *L. quinquestriatus* (Omran 2003). The present study was conducted to examine the cytotoxic activity and mechanism of action of the Egyptian scorpion venom *A. amoreuxi* on the Ehrlich ascites carcinoma (EAC) cells and human breast cancer MCF-7 cell line using both in vitro and in vivo approaches.

## Methods

### Scorpion venom collection and preparation

About 200 specimens of *A. amoreuxi* scorpions were captured from the Western Mediterranean Costal Desert (Alexandria, Egypt) in August 2010. Scorpions were kept alive in separate plastic containers and fed with cockroaches and received water ad libitum. The crude venom was collected using electrical stimulation (12–16 V, 3 ms) of the scorpion telson according to the method described by Abdel-Rahman et al. (2013). The pooled venom (collected from different scorpion specimens) was dissolved in distilled water and centrifuged for 10 min at 5000 rpm to remove cellular debris. Then, supernatant was immediately freeze dried (Labconco freeze dry system, model 77500, USA) and stored at −20 °C until use.

### Experimental animals, ethics and cancer cell lines

Swiss Webster albino mice (20–25 g) were housed in plastic cages (ten animals/cage) under standard laboratory conditions (27 ± 2 °C; 70–80 % humidity; 12-h light/darkness cycle) with standard pellet diet and water ad libitum. The animals were handled in accordance with current guidelines for the care of laboratory animals and ethical guidelines for the investigation of experimental pain in conscious animals. All efforts were made to minimize the number of animals used and their suffering. The first inoculum of EAC and MCF-7 cell lines was purchased from the National Cancer Institute, and VACSERA (Cairo, Egypt), respectively. EAC cells were propagated in the peritoneal cavity of mice through serial intraperitoneal (IP) transplantation of 10^6^ cells (in 0.2 ml of PBS/animal) and transferred every 7 days to new animals. Mice were monitored daily and cell viability was evaluated using trypan blue dye exclusion method (Bincoletto et al. 2005).

### Determination of LD_50_

The approximate median lethal dose (LD_50_) of *A. amoreuxi* venom was estimated using a limited number of experimental animals. Briefly, eight weighted mice were treated with IP injection with different doses (D) of scorpion venom. Then, the animals were monitored for 24 h and the survival time (T) was recorded in each case. The regression line was generated using the data of D/T versus D and LD_50_ was calculated following the equations described in Meier and Theakston (1986). The approximate LD_50_ of *A. amoreuxi* venom was calculated to be 1.1 mg/kg.

### In vitro experiments

#### Cell survival assay

The in vitro cytotoxic effect of scorpion venom was conducted using MCF-7 cell line. Cell survival was determined using sulphorhodamine B (SRB, Biotium, USA) method as previously described (Skehan et al. 1990). Cells were seeded in 96 well microtiter plates at a concentration of 1000–2000 cells/well, 100 μl/well. After 24 h, cells were incubated for 24, 48 and 72 h with various concentrations of *A. amoreuxi* venom (0, 0.01, 0.1, 1, 10, 100 μg/ml). After 72 h from venom application, cells were fixed with 10 % trichloroacetic acid (150 μl/well for 1 h at 4 °C). Cells washed three times using water and stained for 10–30 min at room temperature with 0.4 % SRB dissolved in 1 % acetic acid (70 μl/well). The unbounded dye was removed with 1 % acetic acid. The plates were air dried for 24 h, the dye was solubilized with 10 mM tris base of (pH 7.4, 150 μl/well) for 5 min on a shaker at 1600 rpm. The optical density (OD) was measured spectrophotometrically at 540 nm using the Bio-Rad ELISA microplate reader (Model 550, Japan). The value of IC_50_ was calculated using sigmoidal concentration response curve fitting models (Sigma plot software).

#### DNA extraction and fragmentation analysis

DNA was extracted from both control and treated MCF-7 cells and fragmentation assay was performed (Giri et al. 2003, Peitsch et al. 1993). Cells were lysed using 10 % sodium dodecyl sulfate (SDS) for 30 min. Potassium acetate (8 M) was added to the supernatant and incubated for 1 h at 4 °C. After spinning at 7000 rpm, an equal volume of distilled phenol, chloroform and isoamyl alcohol (25:24:1) mixture was added. The mixture was subjected to centrifugation for 30 min at 3000 rpm, 20 µl/ml of RNase was added and incubated for 30 min at 37 °C. Two volumes of chilled ethanol was added and allowed to stand at 20 °C overnight. The solution was centrifuged (10,000 rpm for 1 h at 4 °C) and the pellet obtained was dissolved in Tris–EDTA (TE) buffer. DNA was estimated by recording the absorbance at 260 nm and 280 nm. DNA was electrophoresed on 2 % agarose gel at 50 V using Tris–borate–EDTA (TBE) buffer. DNA was visualized by incorporation of ethidium bromide (1 mg/ml) in the gel during casting and viewed under UV illumination (302 nm). Documentation was done using BIO-DOC IT™ system.

### In vivo experiments

#### Ehrlich ascites carcinoma model and animal groups

In order to examine in vivo anticancer efficacy of scorpion venom, 80 female albino mice were inoculated with i.p. injection of 10^6^ EAC cells in a volume of 0.2 ml. These mice were randomly divided into five groups (10 or 20 mice/group). After 48 h of tumor inoculation, mice were treated i.p. with *A. amoreuxi* venom or cisplatin every other day according to the following scheme: Group I: normal mice saline-control group (normal control): ten normal mice were injected i.p. with 0.2 ml physiological saline (0.9 g/dL) every other day for 13 days. Group II: EAC-bearing mice control group (EAC control): 20 EAC-bearing mice injected with 0.2 ml physiological saline every other day for 13 days. Group III: EAC-bearing mice venom-treated group: 20 EAC-bearing mice treated with *A. amoreuxi* venom (1/5 LD_50_; 0.22 mg/kg) every other day for 13 days. Group IV: EAC-bearing mice cisplatin-treated group (positive control): 20 EAC-bearing mice treated with cisplatin (0.25 mg/kg; Santos et al. 2011) every other day for 13 days. Group V: *A. amoreuxi* venom-treated group: ten normal mice treated with 1/5 LD_50_ (0.22 mg/kg) of *A. amoreuxi* venom every other day for 13 days. The animals with ascitic tumor were weighed every 3 days. Total experimental period was 25 days and at 14th day, 6 mice in each group were anesthetized and sacrificed to (1) evaluate anti-tumor activity of scorpion venom and (2) to conduct hematological, biochemical and histopathological assay. The remaining mice in Group II–Group IV (n = 14 mice/each group) were kept alive to estimate the mean survival time (MST) and percent increase in life span (% ILS).

##### Collection of ascitic fluid

The ascitic fluid from control and treated groups was collected for the immediate determination of (1) ascites tumor volume; (2) packed cell volume (PCV) and (3) total count/viability of Ehrlich ascites tumor cells (EAT) using hemocytometer and dye-exclusion technique, respectively (Nicol and Prasad 2006). Viability percentage of EAT cells was calculated according to the following equation: (No. of viable/No. of viable and dead cells) × 100.

##### Calculation of MST and percentage of ILS, T/C and TIR

MST of each group was monitored by recording the mortality daily. The end point of experiment was determined by the spontaneous death of animals and MST was calculated according to the equation: MST = (day of first death + day of last death)/2. The percentage of ILS was calculated using equation of Nicol and Prasad (2006) (ILS% = (T − C)/C × 100), where T represents MST of treated animals and C represents MST of the control group. T/C% (treated vs. EAC control) was calculated as MST of treated animals/MST of control group. TIR% (tumor-growth inhibition rate) = (C − T)/C × 100, where T represents mean tumor volume of treated group and C represents mean tumor volume of the control group. According to the criteria of National Cancer Institute (NCI), T/C exceeding 125 % and ILS exceeding 25 % indicate that the drug has a significant anti-tumor activity (Plowman et al. 1997).

##### Hematological and biochemical assays

Blood was withdrawn on day 14 by cardiac puncture method from all animal groups in tubes containing EDTA (for hematological assays) and without anticoagulant (for serum biochemical analysis) in accordance with the method of Frankenberg (1979). Hemoglobin (Hb) and count of white blood cells (WBCs), red blood cells (RBCs) and differential leukocyte were analyzed using standard automated procedures. Biochemical parameters of creatinine, aspartate aminotransferase (AST) and alkaline phosphatase (ALP) were measured in serum using commercial kits (Biodiagnostic Company for Laboratory Services). The levels of liver (see “Tissue collection and histopathological examination” section) glutathione (GSH, non-enzymatic antioxidant), malondialdehyde (MDA, end product of lipid peroxidation) and protein carbonyl content (PCC, a marker of protein oxidation) were determined according to Beutler et al. (1963), Ohkawa et al. (1979) and Levine et al. (1994), respectively.

##### Tissue collection and histopathological examination

Liver and kidney were immediately excised after animal decapitation and rinsed in saline. Small portions from liver and kidney were preserved in 10 % neutral buffered formalin for histopathological examinations according to Bancroft et al. (1996). Hematoxylin and eosin (H&E) stained sections were examined by a light microscope and photographed. The rest of liver tissues were homogenized for biochemical analysis.

#### Ehrlich solid tumor model

Forty female albino mice were randomly divided into four groups (ten mice/group), Group I was kept as control while solid tumor was induced in the other three groups (Group II, III and IV) by subcutaneous (s.c.) inoculation of 0.2 ml of EAT cells (1 × 10^6^ cells/mouse) into the thigh of the lower limb (Mohamed et al. 2003). After 1 week of tumor inoculation, mice were intraperitoneally treated with *A. amoreuxi* venom or cisplatin every other day according to the following scheme. Group II: ten tumorized mice injected with 0.2 ml of PBS every other day for 30 days. Group III: ten tumorized mice treated with 1/5 LD_50_*A. amoreuxi* venom (0.22 mg/kg) every other day for 30 days. Group IV: (positive control) ten tumorized mice treated with cisplatin (0.25 mg/kg, Santos et al. 2011) every other day for 30 days. Tumor mass was measured from the 5th day of tumor inoculation. The measurement was carried out every 5 days for a period of 30 days. The volume of solid tumor was gauged using Vernier caliper (after shaving the tumor bearing thigh of each animal) and was calculated using the formula of A × B^2^ × 0.5, where A and B are the longest and the shortest diameter of tumor, respectively (Orsolic et al. 2006). Five mice from each group were sacrificed on day 31 and tumor mass was removed, weighed, tabulated and preserved in buffered formalin and processed for immunohistochemical (IHC) investigations of the tumor markers KI67, caspase3 and VEGF (markers of proliferation, apoptosis and angiogenesis, respectively).

##### Immunohistochemical analysis of solid tumors

Ehrlich solid tumors were harvested, fixed in formalin and paraffin embedded for IHC analysis (Jakob et al. 2008). After antigen retrieval with 10 mM sodium citrate buffer (pH 6.0) at 80 °C for 10 min, endogenous peroxidases were blocked by 3 % hydrogen peroxide in PBS for 10 min. The slides were then incubated overnight with primary antibodies against Ki67, caspase-3 and VEGF at 4 °C in a humidified chamber and then incubated with horseradish peroxidase (HRP)-conjugated secondary antibodies at 1:100 dilutions for 30 min at 37 °C and visualized by 3,3′-diaminobenzidine tetra hydrochloride reagent (Broad spectrum LAB-SA Detection System from Invitrogen Cat. No. 95-9943-B). The sections were counterstained with hematoxylin and digitally imaged. For IHC quantification, one thousand cells from at least four separate tissue sections were counted (no. of animals per group = 3) using image J software (version 1.48, 32 bit). The number of positive brown-stained cells over the total number of cells was estimated and used to determine the percentage (%) of staining area for each tumor marker.

#### Statistical analysis

Statistical analysis of data was evaluated by one-way ANOVA followed by Tukey–Kramer test (for multiple comparison) to compare between control and treated groups. P values <0.05 were considered as indicative of significance for all comparisons made. All statistical analyses were performed using Sigmaplot statistical software package version 11.

## Results

### In vitro cytotoxic effect of *A. amoreuxi* venom

**Fig. 1:**
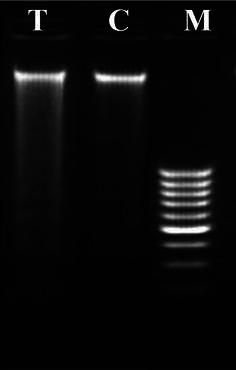
Genomic DNA fragmentation analysis of MCF-7 cells after treatment with the scorpion venom *A. amoreuxi*. DNA was resolved on a 2 % agarose gel and visualized with ethidium bromide. *Lane T* treated cells with 0.5 µg/ml of scorpion venom for 24 h, *lane C* untreated cells (control), *lane M* molecular weight marker (1 kb ladder)

The short-term in vitro cytotoxicity study showed that the IC_50_ value of *A. amoreuxi* venom on MCF-7 cell line was 0.61 μg/ml. In an attempt to check whether the death of MCF-7 cells that occurred as a consequence of *A. amoreuxi* venom treatment was due to apoptosis, DNA fragmentation analysis was performed. DNA was extracted from both control and treated cells after 24 h. The data in Fig. 1 revealed that scorpion venom induced DNA fragmentation when compared to control cells.

### Effect of *A. amoreuxi* venom on tumor growth and survival of EAC-bearing mice

**Fig. 2:**
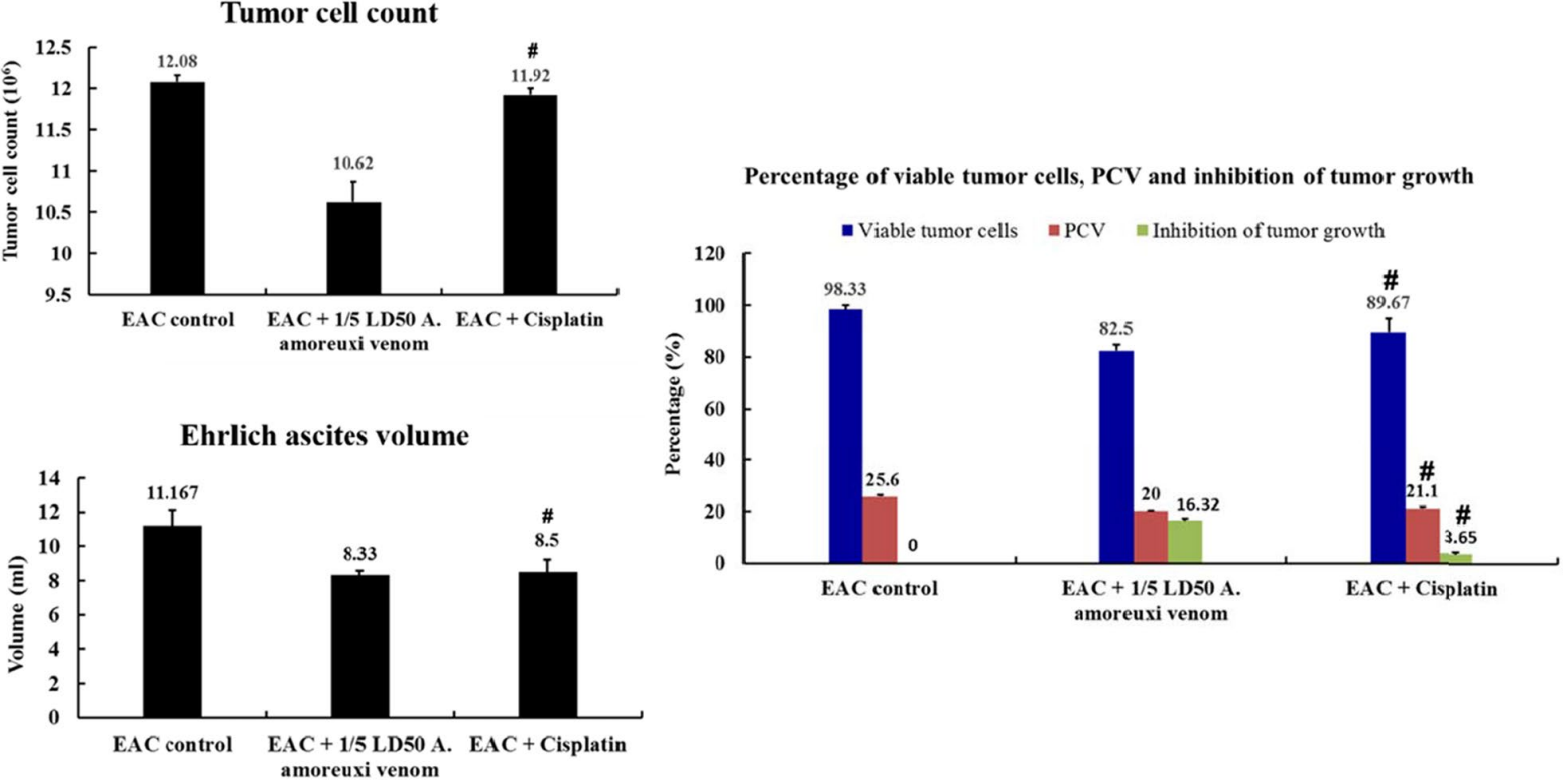
Effect of *A. amoreuxi* venom on tumor growth (tumor cell count and viability, ascites volume and PCV) of EAC-bearing mice. *Hash symbol* represents a significant difference between the three animal groups (EAC control, EAC + 1/5 LD_50_ *A. amoreuxi* venom and EAC + cisplatin) using one-way ANOVA (P ≤ 0.05) followed by a Tukey post hoc test for multiple comparisons

Treatment of EAC-bearing mice with 1/5 LD_50_ of *A. amoreuxi* venom (0.22 mg/kg) significantly reduced (P < 0.05) the increases in the mouse body weight, ascitic volume, PCV, total tumor cell counts and numbers of viable tumor cells in EAC-bearing mice as compared to non-treated animals (Table 1; Fig. 2). These results indicate that *A. amoreuxi* venom possesses significant anti-tumor effects which reached 16.32 %. On the other hand, the percentage of non-viable tumor cells (17.5 %), MST (19.5 days), ILS (25.8 %), the percentage of T/C (125.81 %), and tumor growth inhibition (TIR%, 25.41 %) of scorpion venom-treated mice were increased as compared to control tumor-bearing mice (Table 1; Fig. 2).

**Table 1:** Effect of *A. amoreuxi* venom on body weight and survival time of EAC-bearing mice

	Body weight (g)		Survival time range (days)	MST (days)	ILS (%)	T/C (%)	TIR (%)
	1st day	12th day					
EAC control	21.20 ± 2.41	31.62 ± 3.21	12–19	15.5	–	–	–
EAC + 1/5 LD_50_ *A. amoreuxi* venom	20.21 ± 1.84	28.77 ± 2.33	16–23	19.5	25.80*	125.81*	25.41
EAC + cisplatin (0.25 mg/kg)	21.48 ± 2.19	28.48 ± 3.76	16–25	20.5	32.25*	132.26*	23.88

Data are presented as mean **±** SEM (six animals/group)

* Significant difference between EAC control and treated groups using student unpaired t test (*P* < 0.05)

### Influence of *A. amoreuxi* venom on hematological and biochemical parameters of EAC-bearing mice

**Fig. 3:**
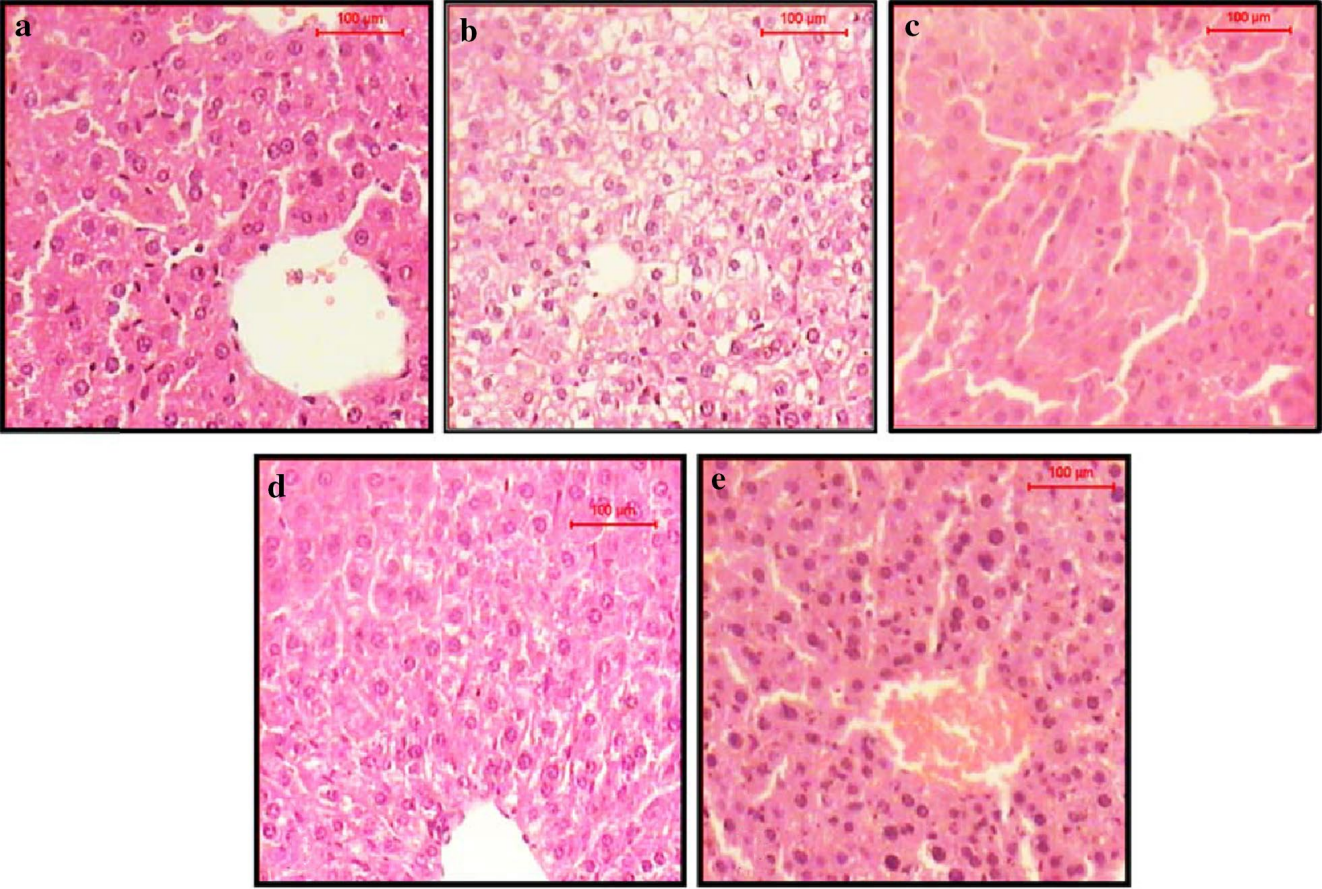
TS of liver of **a** normal control mice shows the normal architecture of hepatic lobule. The central vein surrounded by cords of hepatocytes. Between the strands of hepatocytes the narrow blood sinusoids are often seen, **b** EAC control mice shows hydropic degeneration of the hepatocytes, loss of cell boundaries and ballooning degeneration. Some other hepatocytes showed nuclear pyknosis and karyolysis, **c** 1/5 LD_50_ of venom-treated EAC-bearing mice shows the hepatic lobule that appear more or less like normal, **d** cisplatin-treated EAC-bearing mice shows the hepatic lobule that appear more or less like normal, some hepatocytes shows hydropic degeneration and ballooning degeneration, **e** 1/5 LD_50_ of venom, shows the hepatic lobule that appear more or less like normal. Notice the enlargement and hyperchromasia in the nuclei of the hepatocytes (H&E, *scale bar* 100 µm, magnification ×400)

**Fig. 4:**
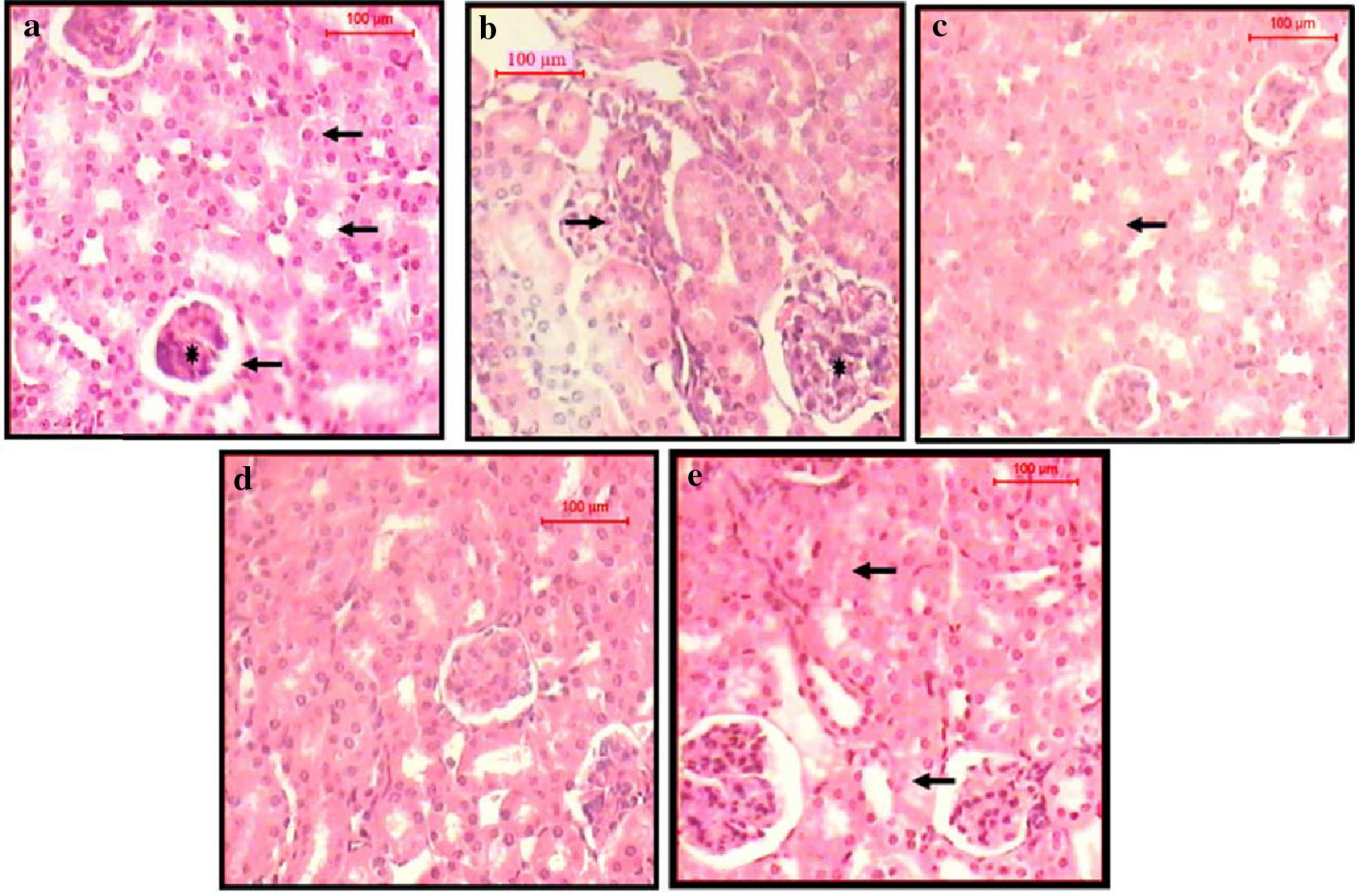
TS of the cortical tissue of the kidney of **a** normal control mice shows renal corpuscle and renal tubules, proximal convoluted tubules (*short arrow*) and distal convoluted tubules (*long arrow*). Notice the glomerulus (*asterisk*) and urinary space (*arrowhead*), **b** EAC control mice shows inflammatory infiltration in the interstitial spaces (*arrow*). The renal corpuscles show congestion and hypercellularity (*asterisk*). The renal tubules exhibit almost normal structure, **c** 1/5 LD_50_ of venom treated EAC-bearing mice shows the renal corpuscle and tubules appear near normal structure, **d** Cisplatin-treated EAC-bearing mice shows the renal corpuscles and tubules that appear near normal structure, **e** 1/5 LD_50_ of venom shows degeneration of some renal tubules (*arrow*) (H&E, *scale bar* 100 µm, magnification ×400)

The data in Tables 2 and 3 showed that treatment with *A. amoreuxi* venom ameliorated EAC-induced alteration in hematological and biochemical parameters, including, RBCs and WBCs count, activities of AST and ALP. Treatment with venom significantly decreased the elevated levels of PCC (a biomarker for protein oxidation) and MDA (a biomarker for lipid peroxidation) and restored the level of GSH in EAC-bearing mice. These results were supported by histopathological sections of liver (Fig. 3) and kidney (Fig. 4), where sections of liver and kidney demonstrated a protective effect of *A. amoreuxi* venom to overcome the deleterious changes resulted from tumor development.

**Table 2:** Effect of *A. amoreuxi* venom on hematological parameters of EAC-bearing mice

	RBCs count (×10^6^/µl)	Haemoglobin (%) (g/dl)	WBCs count (×10^3^/µl)	Neutrophil (%)	Lymphocyte (%)	Monocyte (%)
Normal control	4.06 ± 0.18	6.08 ± 0.15	2.31 ± 0.29	45.06 ± 0.39	50.48 ± 0.57	3.58 ± 0.29
EAC control	2.44 ± 0.18	3.83 ± 0.16	3.23 ± 0.37	57.43 ± 0.42	38.53 ± 0.43	3.31 ± 0.15
EAC + 1/5 LD_50_ *A. amoreuxi* venom	3.96 ± 0.0.8	6.03 ± 0.12	1.86 ± 0.23	45.41 ± 74	50.66 ± 0.84	3.21 ± 0.16
EAC + cisplatin (0.25 mg/kg)	3.23 ± 0.42	5.60 ± 0.62	3.11 ± 0.10	60.73 ± 3.14	38.75 ± 2.65	3.50 ± 0.18
1/5 LD_50_ *A. amoreuxi* venom	4.59 ± 0.08^#^	6.38 ± 0.32^#^	2.71 ± 0.26^#^	44.23 ± 1.73^#^	51.55 ± 1.76^#^	3.36 ± 0.20

Data are presented as mean **±** SEM (six animals/group)

^#^Significant difference between animal groups (normal control EAC control, EAC + 1/5 LD_50_ *A. amoreuxi* venom, EAC + cisplatin and 1/5 LD_50_ *A. amoreuxi* venom) using one-way ANOVA (P ≤ 0.05) followed by a Tukey post hoc test for multiple comparisons

**Table 3:** Effect of *A. amoreuxi* venom on biochemical parameters in serum and liver of EAC-bearing mice

	AST	ALP	Creatinine	GSH	MDA	PCC
Normal control	68.27 ± 0.91	82.74 ± 1.52	0.81 ± 0.07	711.07 ± 2.20	37.80 ± 0.40	2.26 ± 0.43
EAC control	123.82 ± 1.10	165.30 ± 1.14	1.58 ± 0.11	668.94 ± 1.51	45.47 ± 1.28	5.16 ± 0.74
EAC + 1/5 LD_50_ *A. amoreuxi venom*	85.08 ± 0.79	90.02 ± 1.30	1.54 ± 0.05	729.90 ± 1.10	32.48 ± 0.41	4.23 ± 0.61
EAC + cisplatin (0.25 mg/kg)	69.34 ± 0.86	99.20 ± 1.73	1.47 ± 0.03	750.98 ± 0.94	35.24 ± 1.01	4.90 ± 0.66
1/5 LD_50_ *A. amoreuxi venom*	77.13 ± 1.58^#^	135.71 ± 1.02^#^	1.51 ± 0.02^#^	702.00 ± 7.89^#^	35.08 ± 4.06^**#**^	3.99 ± 0.24^**#**^

Data are presented as mean **±** SEM (six animals/group)

^#^Significant difference between animal groups (normal control EAC control, EAC + 1/5 LD_50_ *A. amoreuxi* venom, EAC + cisplatin and 1/5 LD_50_ *A. amoreuxi* venom) using one-way ANOVA (P ≤ 0.05) followed by a Tukey post hoc test for multiple comparisons

### Effect of scorpion venom on solid tumor

**Fig. 5:**
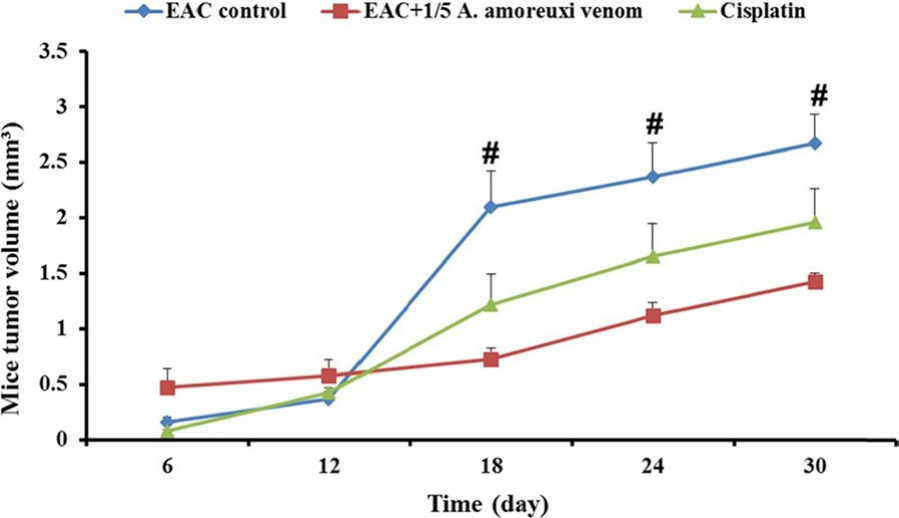
Effect of *A. amoreuxi* venom on solid tumor volume (mm^3^) of EAC-bearing mice. Data are presented as mean ± SEM (five animals/group). *Hash symbol* represents a significant difference between the three animal groups (EAC control, EAC + 1/5 LD_50_ *A. amoreuxi* venom and EAC + cisplatin) at different time intervals (6, 12, 18, 24 and 30 days) using one-way ANOVA (P ≤ 0.05) followed by a Tukey post hoc test for multiple comparisons

**Fig. 6:**
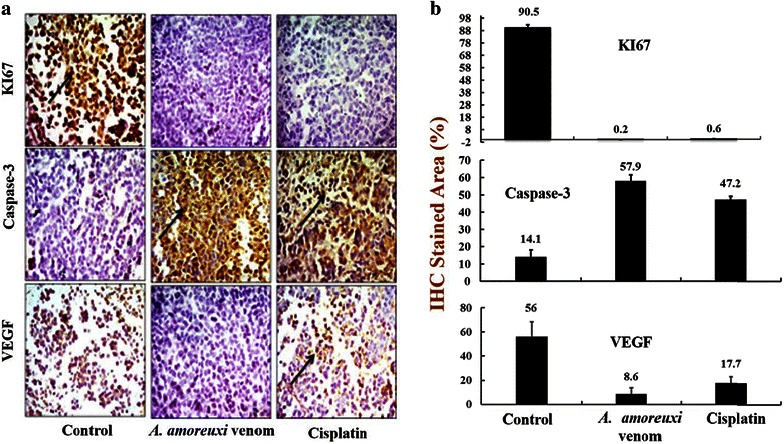
**a** Immunohistochemistry of KI67, caspase-3 and VEGF in tumor sections. Tumorized mice were treated with *A. amoreuxi* venom (1/5 LD_50_) and cisplatin every other day for 30 days then sacrificed and solid tumor was excised to processed for IHC studies. Randomly selected areas from each tumor were analyzed. *Arrowheads* indicate immunohistochemical staining of KI67, caspase-3 and VEGF (magnification: ×400). **b** Comparison between the stained areas of the three tumor markers KI67, caspase-3 and VEGF using Image J

In solid tumor, the average tumor volume in mice treated with *A. amoreuxi* venom every other day for 30 days was markedly (P < 0.05) decreased (1.43 ± 0.07 mm^3^) when compared to that of control mice (2.67 ± 0.26 mm^3^; Fig. 5), reaching about −46.4 %. Quantitatively the weight of tumor lumps extracted from mice treated with scorpion venom was smaller than those of control mice. In order to explore mode of action of *A. amoreuxi* venom on solid tumor, IHC analysis of Ki67, caspase-3 and VEGF expression in Ehrlich solid tumor tissues harvested on day 30 was evaluated. It was found that a significant (P < 0.05) inhibition in KI67 expression was observed in solid tumors from mice treated with *A. amoreuxi* venom as compared to control mice. Also, treatment with *A. amoreuxi* venom significantly enhanced the expression of the apoptotic molecule caspase-3 in tumor tissues. Furthermore, scorpion venom down-regulated the expression of VEGF in tumor tissues, indicating that *A. amoreuxi* venom is able to inhibit process of neovascularization (Fig. 6).

## Discussion

The present study revealed cytotoxic/anti-tumor properties of *A. amoreuxi* on cancer cell lines (MCF-7) and on experimental animal tumor models (Ehrlich ascites and solid tumors). Treatment with *A. amoreuxi* venom showed anti-cancer activity both in vitro or in vivo settings. The toxicity of the venom on cancer cells may be related to its capability to induce necrosis or apoptosis in cancer cells. Several previous studies reported necrotizing and apoptotic effects of scorpion venom peptides on different cancer cell lines. Almaaytah et al. (2012) characterized the cytolytic peptides AamAP1 and AamAP2 (18 amino acids each) from the venom of North African scorpion *A. amoreuxi*. They found that the natural peptides AamAP1 and AamAP2 showed moderate antiproliferative activity against the examined cell lines (LNCaP, U251, PC3 and HMEC-1). While the synthetic analog AamAP-S1 showed higher anticancer efficacy through inducing necrosis in PC3 prostate cancer cell line. The venom peptide Acra3 (66 amino acids) from the Turkish scorpion *A. crassicauda* induced marked cytotoxic effect (IC_50_ value of 5 µg/ml) on mouse brain tumor cells (BC3H1) through both necrotic and apoptotic pathways (Caliskan et al. 2013). Also, previous studies described various necrotic and apoptotic manifestations following treatment with scorpion venoms on different cancer cell lines such as massive releasing of LDH, up-regulation of caspase-3 activity, membrane blebbing, chromatin condensation and DNA degradation (Li et al. 1997; Gupta et al. 2007; Gomes et al. 2010; Zargan et al. 2011a, b, c). In this regard, our finding (DNA fragmentation in treated cells) may give an indication about the apoptotic potency of *A. amoreuxi* venom on MCF-7 cells. Interestingly, these results were further supported by enhanced expression of caspase-3 in Ehrlich solid tumor tissues following scorpion venom treatment.

Ascites fluid constitutes an essential nutritional source for tumor cells growing and development (Gupta et al. 2004). Our data showed that IP administration of *A. amoreuxi* venom significantly reduced the ascites (tumor volume), tumor cell count, and increased the percentage of trypan blue positive stained dead cells in tumor-bearing mice. The reduction in tumor volume could be attributed to the treatment of *A. amoreuxi* venom (1) inhibited the proliferation of EAT cells; (2) enhanced apoptotic pathways of cancer cells and (3) direct necrotizing effect of scorpion venom phospholipase A2/cytolytic peptides (Almaaytah et al. 2012). Consequently, *A. amoreuxi* venom treatment significantly increased MST and %ILS as compared to EAC control group. Life span prolongation (>25 %) is directly related to the decrease in viable cell count and ascitic fluid accumulation. It is well known that prolongation of life span is one of the important criteria to evaluate potency of anticancer drugs. According to the criteria of National Cancer Institute, T/C exceeding 125 % and ILS exceeding 25 % indicate that the drug has a significant anti-tumor activity (Plowman et al. 1997; Gupta et al. 2004; Agrawal et al. 2011).

Myelosuppression and anemia (reduced hemoglobin) have been frequently observed in ascites carcinoma (Maseki et al. 1981). Anemia encountered in ascites carcinoma is mainly due to iron deficiency, either by hemolytic or myelopathic conditions, which finally lead to reduced RBCs’ count (Sreelatha et al. 2011). In the present study, elevated WBCs’ count, reduced hemoglobin and RBCs’ count were observed in EAC control mice, where administration of *A. amoreuxi* venom restored hemoglobin level and maintained normal values of RBCs and WBCs. The result of total differential leucocytes count revealed that neutrophils increased within EAC-bearing mice might be due to the acute inflammatory response or stress due to proliferation of EAT cells (Hashem et al. 2004). While neutrophils present in EAC-bearing mice treated with *A. amoreuxi* venom might be decreased as a result of immunostimulating effect of scorpion venom and defence of the host against tumor cells. The reversal of hematological parameters indicates that this venom may possess protective action on the hematopoietic system without inducing myelotoxicity. For 14 days, *A. amoreuxi* venom did not exhibit any adverse effect (Sreelatha et al. 2011). Regarding the elevated liver enzymes (ALP and AST) in EAC-bearing mice, lack of hepatic toxicity was observed upon treatment with *A. amoreuxi* venom, indicating that the venom has a protective effect against organ dysfunction and cellular injury of liver. These results were further supported by liver histopathology. Recently, Bekkari and Laraba-Djebari (2015) reported the beneficial effects of *A. australis* venom which partially restored the hepatic alteration induced by the carcinogenic agent of fumonisin (FB1 mycotoxin). Moreover, reducing extent of oxidative stress may explain how *A. amoreuxi* venom protected liver of treated animals from damage induced by tumor. We propose that treatment with *A. amoreuxi* venom decreased the tumor burden and consequently decrease oxidative stress manifested by restoring levels of MDA, PCC and GSH in liver cells. It was reported that reactive oxygen species (ROS) are probable mediators of cytotoxicity and their role in cancer development, metastasis, progression, and survival is well documented (Cerutti 1994; Sur and Ganguly 1994; Iqbal and Okada 2003; Goswami et al. 2008; Tong et al. 2015). Interestingly, similar results were obtained with the venom of *A. australis* which significantly decreased level of oxidative stress biomarkers in mice hepatic tumor (Bekkari and Laraba-Djebari 2015).

The present results also showed that the tumor weights and volumes of Ehrlich solid tumor were significantly decreased in mice-treated with *A. amoreuxi* venom according to our morphometrical and histological data. Some sort of shrinkage of tumors occurred, and the delay of tumor growth was evident. Inhibition of solid tumor growth by this venom might be as a consequence of cell cycle arrest, apoptosis and/or necrosis of tumor cells or it was the result of activation of immune system by scorpion venom. Inhibition of cell proliferation is considered as a basic parameter in cancer studies (Van Heusden et al. 1997). Uncontrolled cell cycle progression has been considered as a sign of cancer development, and therefore, is a usual target for developing potential anti-cancer drugs (Deep and Agarwal 2008). In this communication, the proliferation index was investigated through evaluating the expression of Ki67 in solid tumors of control and venom-treated mice. The data clearly indicated that *A. amoreuxi* venom inhibited expression of Ki67 in tumor tissues as compared to control animals. Ki-67 is a nuclear protein expressed in proliferating cells (in all phases of the cell cycle except G0) and serves as a good marker for proliferation and may be required for maintaining cell proliferation (Schluter et al. 1993; Miller et al. 1994). Venoms from various scorpions have been reported to prevent propagation of different cell lines such as prostate cancer, human leukemia and neuroblastoma (Gupta et al. 2007; Zhang et al. 2009; Zargan et al. 2011a). For example, venom of *A. crassicauda* inhibited proliferation of human neuroblastoma cell lines through arresting S-phase and induction of apoptosis (Zargan et al. 2011a). Apoptosis is an important pathway in anti-tumor drug response (Makin and Dive 2001; Galeano et al. 2005). Two pathways have been described for cells undergoing apoptosis; (1) death-receptor-induced apoptosis (extrinsic pathway through activation caspases-8, 10 and (2) mitochondria-mediated apoptosis (intrinsic pathway through activation of caspases-9) (Hu and Kavanagh 2003; Franco et al. 2009). Caspase-3 is a key protease necessary for the execution of apoptosis via the activation of caspases-8 leading to DNA damage and cell death (Zargan et al. 2011a). The venom of *A. amoreuxi* enhanced expression of caspase-3 in solid tumor tissues as compared to their control group. This finding was concomitant with our in vitro study and may explain mechanism of DNA fragmentation in MCF-7 cells treated with *A. amoreuxi* venom. Furthermore, *A. amoreuxi* venom revealed anti-angiogenic activity through down-regulation the expression of VEGF in solid tumor. Inhibition of VEGF in tumor is an attractive strategy used to impact angiogenesis-dependent tumor growth and metastasis. Increased VEGF expression is closely associated with an increase in microvessel density (Sadick et al. 2005). Indeed, there are few studies reported capability of scorpion venom peptides to suppress neovascularization and angiogenesis in tumor tissues through decreasing the level of angiogenic factors such as VIII, alpha-SMA, Dll4, Notch1 and VEGF (Lima e Silva et al. 2010; Sun et al. 2011).

## Concluding remarks

*Androctonus amoreuxi* venom exerted cytotoxic effects on tumor cells via anti-proliferative, apoptotic and anti-angiogenic activities. Using recent technologies of venomics (venom proteomics and transcriptomics), further studies are needed towards characterization of the active components from this venom (and other scorpion species inhabiting the Egyptian deserts) and developing them into potential anticancer agents. Moreover, the present study opens the door for detailed investigations about interaction of scorpion venom and conventional antitumor drugs such as cisplatin and others.
